# A Comparative Genome Analysis of PME and PMEI Families Reveals the Evolution of Pectin Metabolism in Plant Cell Walls

**DOI:** 10.1371/journal.pone.0072082

**Published:** 2013-08-12

**Authors:** Maojun Wang, Daojun Yuan, Wenhui Gao, Yang Li, Jiafu Tan, Xianlong Zhang

**Affiliations:** National Key Laboratory of Crop Genetic Improvement, Huazhong Agricultural University, Wuhan, Hubei, China; Iowa State University, United States of America

## Abstract

Pectins are fundamental polysaccharides in the plant primary cell wall. Pectins are synthesized and secreted to cell walls as highly methyl-esterified polymers and then demethyl-esterified by pectin methylesterases (PMEs), which are spatially regulated by pectin methylesterase inhibitors (PMEIs). Although PME and PMEI genes are pivotal in plant cell wall formation, few studies have focused on the evolutionary patterns of the PME and PMEI gene families. In this study, the gene origin, evolution, and expression diversity of these two families were systematically analyzed using 11 representative species, including algae, bryophytes, lycophytes and flowering land plants. The results show that 1) for the two subfamilies (PME and proPME) of PME, the origin of the PME subfamily is consistent with the appearance of pectins in early charophyte cell walls, 2) Whole genome duplication (WGD) and tandem duplication contribute to the expansion of proPME and PMEI families in land plants, 3) Evidence of selection pressure shows that the proPME and PMEI families have rapidly evolved, particularly the PMEI family in vascular plants, and 4) Comparative expression profile analysis of the two families indicates that the eudicot *Arabidopsis* and monocot rice have different expression patterns. In addition, the gene structure and sequence analyses show that the origin of the PMEI domain may be derived from the neofunctionalization of the pro domain after WGD. This study will advance the evolutionary understanding of the PME and PMEI families and plant cell wall development.

## Introduction

Plant cell walls are complex and dynamic structures composed of diverse polysaccharides and proteins, mainly including cellulose, hemicellulose, and pectin [1], and are generally divided into two functional categories: primary walls surrounding the growing cell and secondary walls, which are thickened structures [2,3]. Pectins, a type of polysaccharides, appeared after the divergence of chlorophyta and charophyce [4,5], and are a major component of plant primary cell walls. Pectins are important for contributing to cellular structural integrity, cell adhesion and the mediation of defense responses [6]. These polysaccharides mainly accumulate in primary cell walls and occupy 35% in eudicots and noncommelinid monocots and only 2-10% in grass primary walls [7].

Pectins are assumed to be biosynthesized in *cis*-Golgi by a large number of glycosyltransferases, methyltransferases and acetyltransferases. The basic backbones of pectins are composed of homogalacturonan (HG), xylogalacturonan (XGA), apiogalacturonan, rhamnogalacturonan I (RGI) and rhamnogalacturonan II (RGII) [8]. Homogalacturonan (HG) are methyl-esterified in *medial*-Golgi and transported to the primary cell wall in a highly methyl-esterified state, in which they are demethyl-esterified by pectin methylesterases (PMEs) [9], and spatially regulated by pectin methylesterase inhibitors (PMEIs) [10-12]. The demethyl-esterified pectins can bind Ca^2+^ to form a rigid gel, which plays a critical role in cell wall stiffening [13,14]. Therefore, the methyl-esterification status of HG crucially affects cell wall texture and mechanical properties. In addition, pectins can also be degraded by pectinases [15]. Research on the methyl-esterification of HG in primary walls helps clarify the mechanisms of cellular growth and cell shape modeling [16].

The mature and active region of PME genes mainly consists of the PME domain. In higher plants, the PME genes are classified into two types. Both of the two types of genes possess a PME domain. However, compared to type II PME (subsequently referred to as PME) gene, type I PME (subsequently referred to as proPME) gene possesses an additional pro domain [17,18]. The pro domain is located at the N-terminus of proPME genes and shares similarities with the PMEI domain of the PMEI genes. The function of the pro domain has been reported, including involvement in maintaining unprocessed PME in the Golgi [19]. Because the sequence of the pro domain is similar to the PMEI domain, former studies inferred that it might play a role in auto-inhibitory activity of mature PME proteins (to prevent premature demethoxylation) [20]. However, these studies mainly focused on the function of a few genes using experimental methods, and there were hardly any comprehensive studies regarding how PME and PMEI genes originated. The recent availability of genome sequences for many plant species enables comparative genome analyses to make inferences regarding the origin and evolution of PME and PMEI genes.

The mechanisms underlying the origin of new genes are mainly *de novo* gene birth and the duplication-divergence hypothesis [21-23]. The modes of gene duplication can be summarized as six mechanisms (whole genome duplication (WGD), tandem duplication, proximal duplication, DNA based transposed duplication, retrotransposed duplication and dispersed duplication) [24]. In fact, WGD widely occurs in different species and plays a vital role in organism evolution [25], which often leads to the formation of new species or reflects adaptive evolution when organisms confront ecological stresses [26]. Genome evolution research has detected that the *Arabidopsis* and grape genomes shared an identical γ duplication event. Additionally, *Arabidopsis* experienced two species-specific WGDs (α and β). Both monocot rice and sorghum have experienced two WGDs (σ and ρ) [27,28]. Gene functional divergence, including subfunctionalization, neofunctionalization, pseudogenization and concerted evolution [29], is the direct consequence of gene duplication, which often results in gene family expansion. Study on gene family evolution provides important clues for explaining gene function divergence [30,31].

Currently, the phylogenetic appearance of pectin polymers in cell walls was discovered using chemical approaches, whereas there are no systematic reports of genetic evidence and functional research. To reveal the gene origination of the PME genes and the evolutionary relationship between pro and PMEI domains, we performed a comparative genome analysis of the PME and PMEI families across 11 species, including algae, bryophytes, lycophytes and flowering land plants. The results imply that the origin of PME genes coordinates with the appearance of pectin in cell walls. The PMEI domains may be derived from duplication and divergence of the pro domain and have rapidly evolved. The expression profile analyses of PME and PMEI families show different expression patterns in the model plants *Arabidopsis* and rice. In addition, a network analysis infers that the demethyl-esterification process of pectin conferred by PME and PMEI families is involved in a complex metabolic network. This study provides basic clues for further understanding the relationship between pectin metabolism and plant cell wall evolution.

## Materials and Methods

### Retrieval of PME and PMEI Gene Family Sequences

The gene models of *Arabidopsis thaliana* and *Oryza sativa* were downloaded from TAIR (The *Arabidopsis* Information Resource, http://www.arabidopsis.org/) and RGAP (Rice Genome Annotation Project, http://rice.plantbiology.msu.edu/). The gene files of *Carica papaya*, *Vitis vinifera*, *Populus* *trichocarpa* , *Solanum* *lycopersicum* , *Sorghum* *bicolor* , *Physcomitrella* *patens* and *Selaginella* *moellendorffii* were downloaded from Phytozome (http://www.phytozome.net/) [32]. The gene information of *Amborella* *trichopoda* was downloaded from http://www.amborella.org/ [33]. The gene models of *Coleochaeteorbicula* were downloaded from the National Center for Biotechnology Information (NCBI) GenBank (JO233843-JO252228) [34].

The Hidden Markov Model (HMM) profiles of PF01095 (PME domain) and PF04043 (PMEI domain) were downloaded from PFam database (http://pfam.sanger.ac.uk/), and the HMMER software package [35] was used to detect PME and PMEI genes with the best domain e-value cutoff of 1e-10. These sequences were regarded as potential PME and PMEI genes. To validate the HMM search, these potential sequences were used as queries to search the NCBI non-redundant (nr) protein database with blastp program of GenBank and only the results with the best hits (an e-value less than 1e-5) of “pectin methylesterases” and “pectin methylesterases inhibitor” were retained. Finally, partial genes were manually removed. An in-house Perl script was used to extract the domain sequences with the boundary site information from the HMM results. The domain sequences were further checked manually.

Orthomcl software was used to infer the orthologous genes among the species with the default settings [36], which initially required an all-vs-all blastp, and then the mcl clustering algorithm was used to deduce the relationship between genes. The orthologous genes were defined as genes in a cluster from at least three species.

### Genome Synteny and Gene Duplication

The WGD information of *Arabidopsis* , grape, rice and sorghum was downloaded from former studies [27,28], and the PME and PMEI genes were detected. Tandem duplication genes were identified based on the physical location in the individual chromosome with no more than one intervening gene. To further analyze the genome synteny, the syntenic blocks among *Arabidopsis* , papaya, poplar, grape, tomato, rice and sorghum were downloaded from the Plant Genome Duplication Database (PGDD) [37]. All the PME and PMEI genes were mapped to the syntenic blocks for intra- and inter-genomic comparison. The Circos software was used to draw the syntenic diagram [38].

### Motif Identification and the Exon-intron Structural Analysis

For all the PME, pro and PMEI domain sequences, the online MEME program (version 4.0.0) [39] (http://meme.sdsc.edu/meme/cgi-bin/meme.cgi) was employed to identify and analyze the conserved motifs among amino acid sequences with the following parameters: number of repetitions, any; maximum number of motifs, 10; and optimum motif width set to >6 and <50.

The gene structure information of the PME and PMEI families were parsed from the General Feature Format (GFF) files of every species using an in-house Perl script. The diagrams of the exon-intron structures were drawn using the online program GSDS (http://gsds.cbi.pku.edu.cn/) [40].

### Phylogenetic Analysis

For all the pro and PMEI domains, the sequences were aligned using Clustal X (version 2.0) [41], and the neighbor-joining (NJ) tree was constructed using PHYLIP software [42]. The maximum probability method was used to construct the consensus domain sequence for every species using the HMMER package. The MEGA5 program was then used to construct a maximum-likelihood (ML) tree of the pro and PMEI consensus domain sequences and the PME orthologous genes using the Whelan And Goldman (WAG) model based on the BIC scores (Bayesian Information Criterion) [43,44]. The molecular clock test was performed by comparing the ML value of the orthologous gene topology with and without the molecular clock constraints under the WAG model. The bootstrap value was 500 to construct the phylogenetic tree.

### The Estimation of the Rates of Gene Evolution

The multiple alignment analysis of the protein domain sequence was performed using the Clustal X (version 2.0) [41], and the coding sequences were aligned and guided by alignments of protein sequences using the PAL2NAL software with the NOGAPS parameter [45]. The ratio of nonsynonymous substitutions per nonsynonymous site (Ka) to synonymous substitutions per synonymous site (Ks) (omega) homologous gene pairs was calculated with the yn00 procedure of the PAML package [46]. Based on the definition of Ka/Ks, a value less than 1 indicates negative or purifying selection acting on amino acid changes, whereas a value greater than 1 indicates positive selection, which may indicate adaptive evolution. The saturation effects were excluded by discarding the gene pairs in which Ks >2.5.

### The Expression Analysis of the PME and PMEI families in *Arabidopsis* and Rice

The *Arabidopsis* microarray data were downloaded from the Gene Expression Omnibus database (http://www.ncbi.nlm.nih.gov/geo/) with the GSE series accession numbers GSE5629, GSE5630, GSE5631, GSE5632, GSE5633 and GSE5634. The expression profile data of rice PME and PMEI families were downloaded from the CREP database (http://crep.ncpgr.cn). Subsequent data processing was identical to former research [47].

### Network Assembly and Functional Enrichment

In this study, the *Arabidopsis* PME and PMEI genes were submitted to the Arabidopsis Network Analysis Pipeline (ANAP) [48], which effectively integrated 11 publicly available *Arabidopsis* network databases. The functional enrichment analysis of the genes involved in the PME and PMEI networks were conducted using Blast2GO software with the molecular function category of level three [49].

## Results

### Genome-wide Identification of PME and PMEI Genes

**Figure 1:**
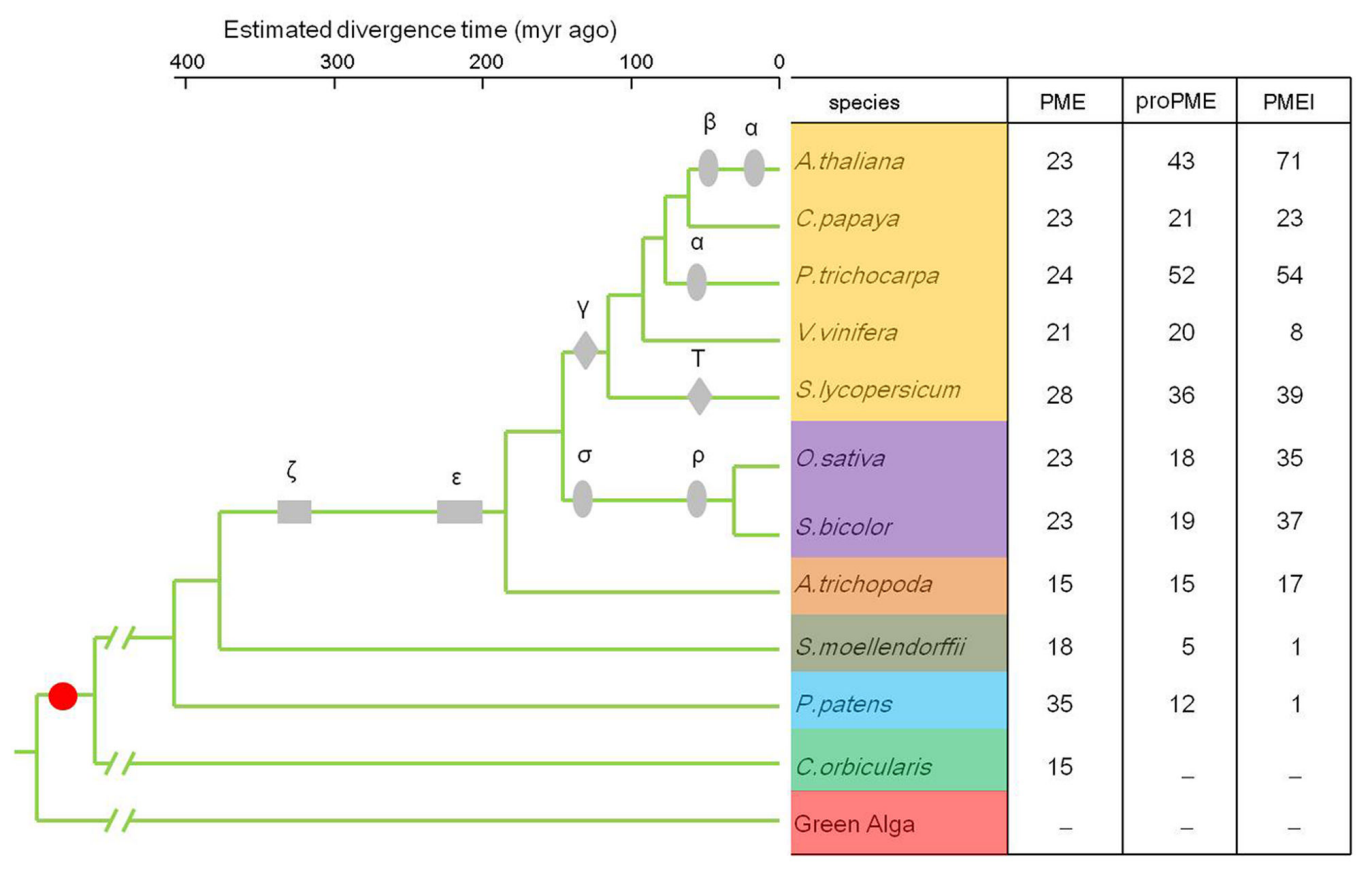
The copy number of PME and PMEI families in the collected species. The ultrametric tree was modified from Jiao et al. [23], Popper et al. [4] and Lee et al. [35]. The grey boxes (ζ, ε), diamonds (γ, T) and ellipses (α, β, σ, ρ) show the whole genome duplication events in the species, the red dot indicates when pectins appeared in the cell wall of organisms. -Indicates not detected.

Through the genome-wide identification of PME and PMEI domains encoding genes, the number of the two families was summarized in Figure 1 and Table S1. The results show that only 15 PME genes exist in *C* *. orbicula* , a representative member of the charophytes that diverged after pectin appeared in the cell walls. There were 35 and 18 PME genes detected in the bryophyte *P* *. patens* and lycophyte *S* *. moellendorffii* , but only 12 and 5 proPME genes and one PMEI gene were detected in the two species, respectively. Furthermore, the results show that these two gene families widely appear in the basal angiosperms *A* *. trichopoda* and also the monocot and eudicot species. The PME family copy numbers in *Arabidopsis* are identical to former research, but 6 more in rice and 13 less in poplar [17], which may be due to the update of genome annotations.

### Gene Family Expansion

The WGD and tandem duplication of the PME and PMEI genes were analyzed to study the gene family expansion. After an in-depth analysis of the WGD information [27,28], 5 PME genes of *Arabidopsis* were derived from the α duplication event, 21 and 7 proPME genes from the α and β events, respectively, 20 and 12 PMEI genes from the α and β events, respectively (Table 1). In grape, 3 PME and 3 proPME genes were derived from the γ duplication event. In the monocot rice, we found that 4 and 7 PME genes, 7 and 9 proPME genes, and 2 and 14 PMEI genes were derived separately from the σ and ρ duplication events, respectively. These results show that the number of original PME genes was relatively conserved, but the species-specific WGD events contributed to the expansion of the proPME subfamily and PMEI family. A gene tandem duplication analysis of the two families showed that 15 of the 71 PMEI genes in *Arabidopsis* were tandem duplication genes, among which we detected a cluster of 7 members in chromosome 5 (AT5G46930, AT5G46940, AT5G46950, AT5G46960, AT5G46970, AT5G46980, and AT5G46990). Although there are 4 more PMEI genes in sorghum than rice, they have a similar number of PME and proPME genes. Through the gene duplication analysis, we found that the proPME and PMEI families rapidly expanded, and possibly, such substantial gene expansion in the land plants was driven by their functional specialization of cell wall formation (Figure 1).

**Table 1:** A summary of the modes of PME, proPME and PMEI gene duplication in *A. thaliana*, *V. vinifera*, *O. sativa* and *S. bicolor*.

Species	Subfamily	Whole Genome Duplication	Tandem Duplication	Other^*^	Total
*A. thaliana*	*PME*	5^α^	2	16	23
	*proPME*	7^β^; 21^α^	6	9	43
	*PMEI*	12^β^; 20^α^	15	24	71
*V. vinifera*	*PME*	3^γ^	2	16	21
	*proPME*	3^γ^	4	13	20
	*PMEI*	-^**^	3	5	8
*O. sativa*	*PME*	4^σ^; 7^ρ^	2	10	23
	*proPME*	7^σ^; 9^ρ^	2	0	18
	*PMEI*	2^σ^; 14^ρ^	6	13	35
*S. bicolor*	*PME*	4^σ^; 10^ρ^	2	7	23
	*proPME*	6^σ^; 11^ρ^	2	0	19
	*PMEI*	4^σ^; 16^ρ^	10	9	39

α and β indicate two recent *Arabidopsis* duplication events, and γ indicates the triplication event that all eudicots shared. σ and ρ indicate the duplications that rice and sorghum shared.

^*^ Gene modes of proximal duplication, DNA-based transposed duplication, retrotransposed duplication and dispersed duplication are included.

^**^ No genes detected in the relative mode.

**Figure 2:**
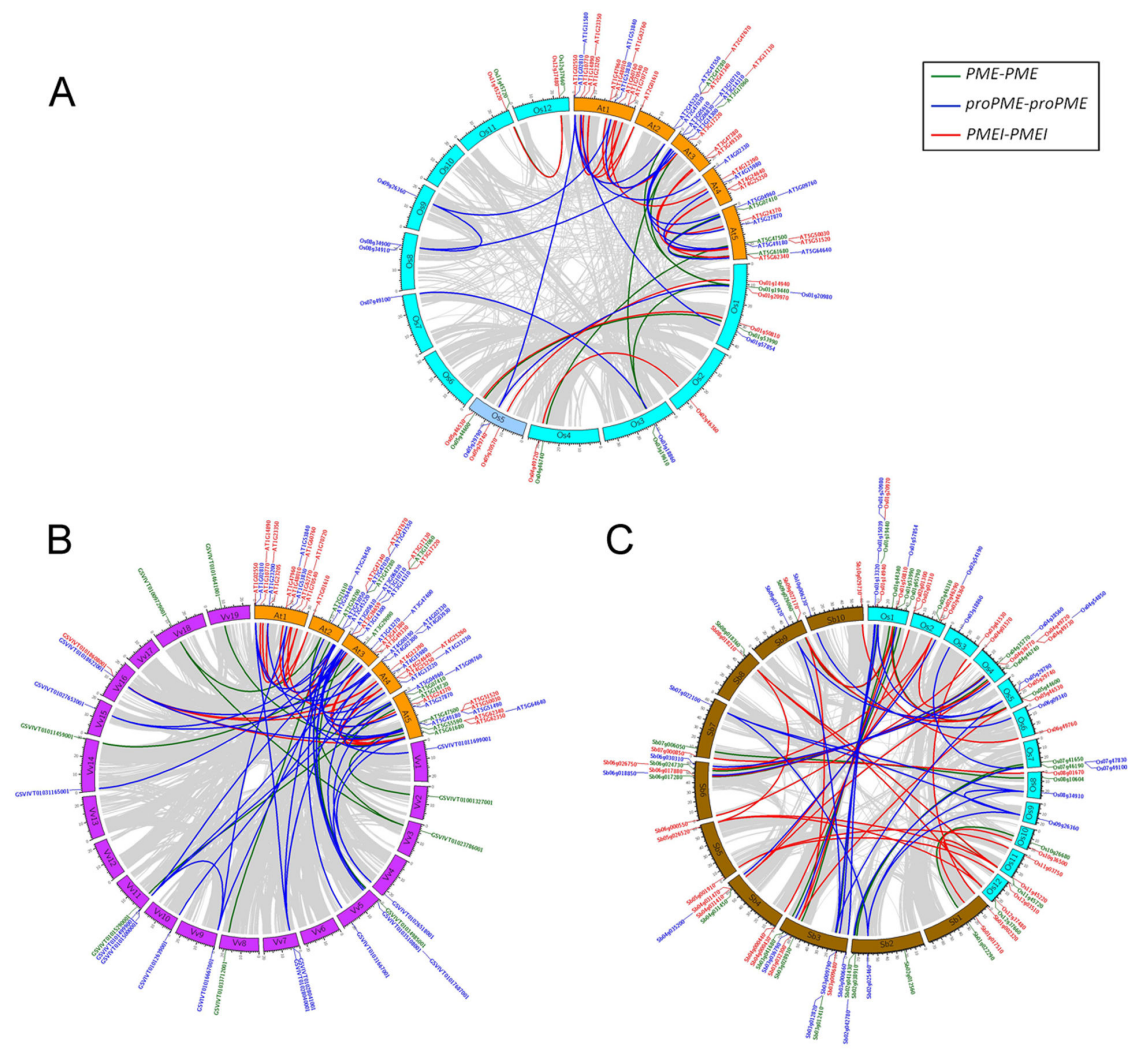
The intra- and inter-genomic comparison analyses showed gene synteny of the PME and PMEI families in *A. thaliana* (At), *V. vinifera* (Vv), *O. sativa* (Os) and *S. bicolor* (Sb). The synteny gene pairs were parsed from the Plant Genome Duplication Database (PGDD). The gray lines indicate whole genome duplication blocks between species, the green lines indicate the synteny of PME genes, the purple lines indicate the synteny of proPME genes, and the red lines indicate the synteny of PMEI genes. (A) gene synteny of PME and PMEI families in *A. thaliana* (At) and *O. sativa* (Os), (B) gene synteny of PME and PMEI families in *A. thaliana* (At) and *V. vinifera* (Vv), and (C) gene synteny of PME and PMEI families in *O. sativa* (Os) and *S. bicolor* (Sb).

Genome synteny of the PME and PMEI families was then analyzed. The results show that there is good synteny through the inter-genomic comparison within the eudicots and monocots, but only a few genes are syntenic between eudicots and monocots (Figure 2), which might be reason of the different ancestral genome organization, because eudicot genomes might originate from 7 chromosomes and monocot genomes might originate from 5 chromosomes [29].

### Exon-intron Structure and Phylogenetic Analysis

**Figure 3:**
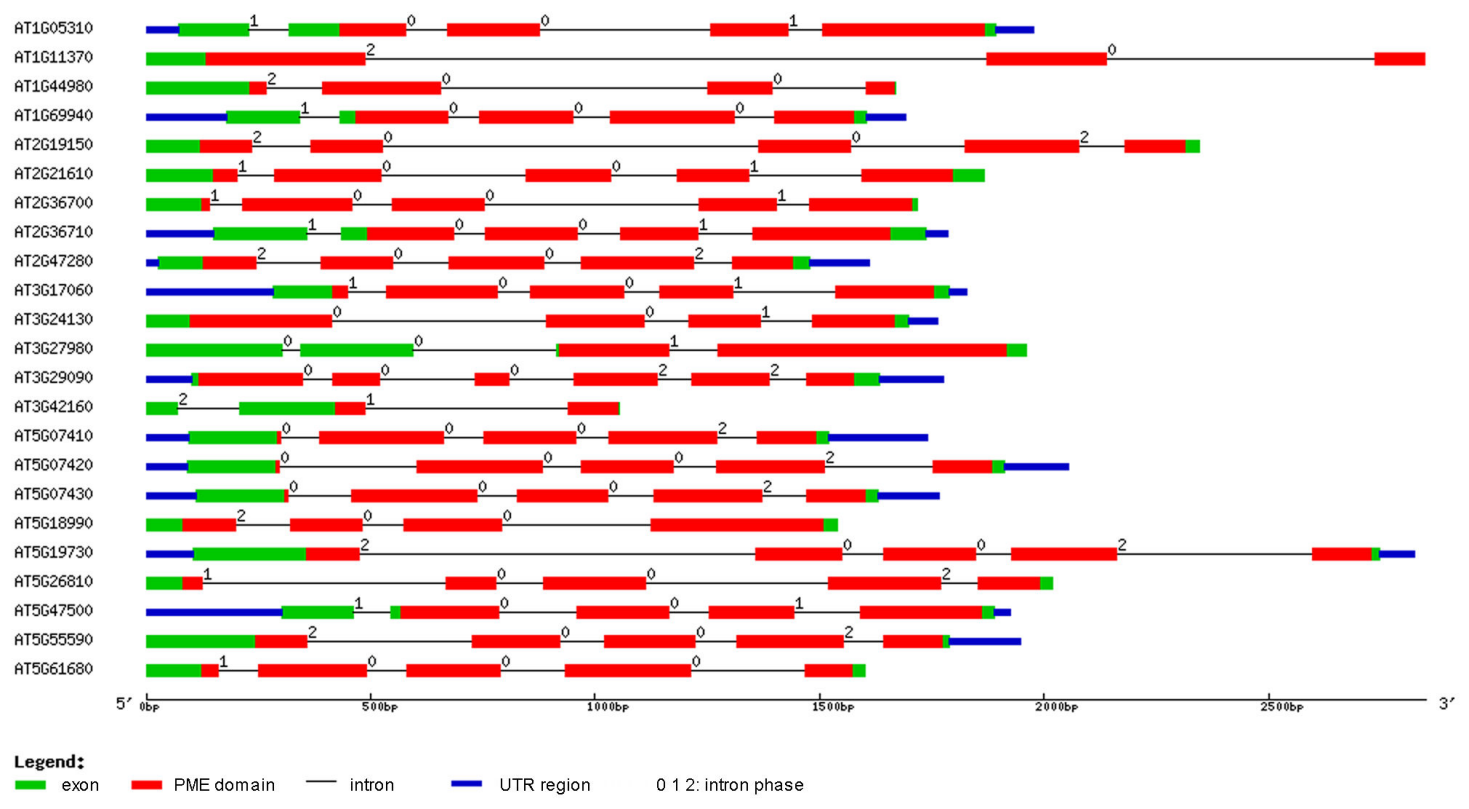
The exon-intron structural analysis of the 23 PME genes in *Arabidopsis* . The gene structures were drawn using the online tool GSDS. The legend shows that the blue boxes are UTR regions, the green boxes are exons, the black lines are introns, the red boxes are the PME domains, and numbers at the exon-intron joints are intron phases.

Gene exon-intron structure analyses of the PME and PMEI families were conducted in 10 species (Table 2). Obviously, the average gene length of proPME is larger than PME. Compared to the PME and proPME genes, the PMEI genes are shortest and the average length is only several hundred base pairs. In addition, most PME genes possess more than 4 exons, except for monocots and the basal angiosperm *A* *. trichopoda* . The proPME genes contain only 2.55 exons on average (from 1.87 to 3.23), but the average exons length of proPME is at least twice that of PME. However, most genes in the PMEI family possess just one exon in all the species. With a graphical display, we depicted the PME gene structures of *Arabidopsis* (Figure 3). The results show that most PME domains of the PME genes consist of 3 exons. Conversely, most of the PME domains of the proPME genes possess only one intron. The pro and PMEI domains possess few introns (Figure S1).

**Table 2:** A summary of gene length, exon number, exon length and intron length of the PME, proPME, and PMEI in representative species.

Species	Average Gene Length				Average Exons per Gene				Average Exon Length				Average Intron Length^a^	
	*PME*	*proPME*	*PMEI*		*PME*	*proPME*	*PMEI*		*PME*	*proPME*	*PMEI*		*PME*	*proPME*
*P* *. patens*	2330	2692	-		4.28	3.23	-		273	546	-		666	688
*S* *. moellendorffii*	1284	2300	-		4.41	3	-		217	579	-		413	338
*A* *. trichopoda*	3159	2860	562		3.73	1.87	1.06		213	658	517		1027	2122
*S. bicolor*	1794	2917	878		3.35	2.05	1.16		327	872	561		667	2122
*O. sativa*	2623	3065	938		3.47	1.89	1.12		322	958	559		868	1709
*S* *. lycopersicum*	2683	3145	686		4	2.58	1.13		257	665	507		524	612
*V. vinifera*	2576	3052	960		4.94	2.93	1.33		248	434	562		558	1124
*P* *. trichocarpa*	2550	2654	885		4.63	2.25	1.09		228	747	556		454	1355
*C. papaya*	2600	2896	851		4.74	3.07	1.15		246	349	616		618	691
*A. thaliana*	1933	2476	817		4.67	2.67	1.25		227	648	470		429	942
Mean Value^b^	2353	2805	822		4.22	2.55	1.16		255	645	543		622	1170

^a^ The intron length of the PMEI genes were not shown because many of them do not have introns.

^b^ The mean values of the gene length, exon number, exon length and intron length in the 10 species.

Indicated only one PMEI gene was detected in *P* *. patens* and *S* *. moellendorffii* , and the structural information was not shown here.

Genome-wide gene identification indicates that angiosperm genomes have a relatively conserved number of PME genes (Figure 1). Domain sequence alignment of the PME genes shows that the PME domains are highly conserved in studied species (Figure S2). Then, Orthomcl software was used to detect the orthologous PME genes in the 11 genomes (Table S2) with the definition that genes in a cluster from at least 3 different species were orthologous. Compared to the species tree in Figure 1, the phylogenetic analysis of a well detected orthologous cluster shows good coordination (Figure S3). Based on the divergence time of the species tree, the divergence of the bryophyte mosses and charophyte *C* *. orbicula* could date back to 470 and 560 million years ago, which was slightly earlier than former results [50]. More accurate estimation should be performed with larger scale molecular data.

**Figure 4:**
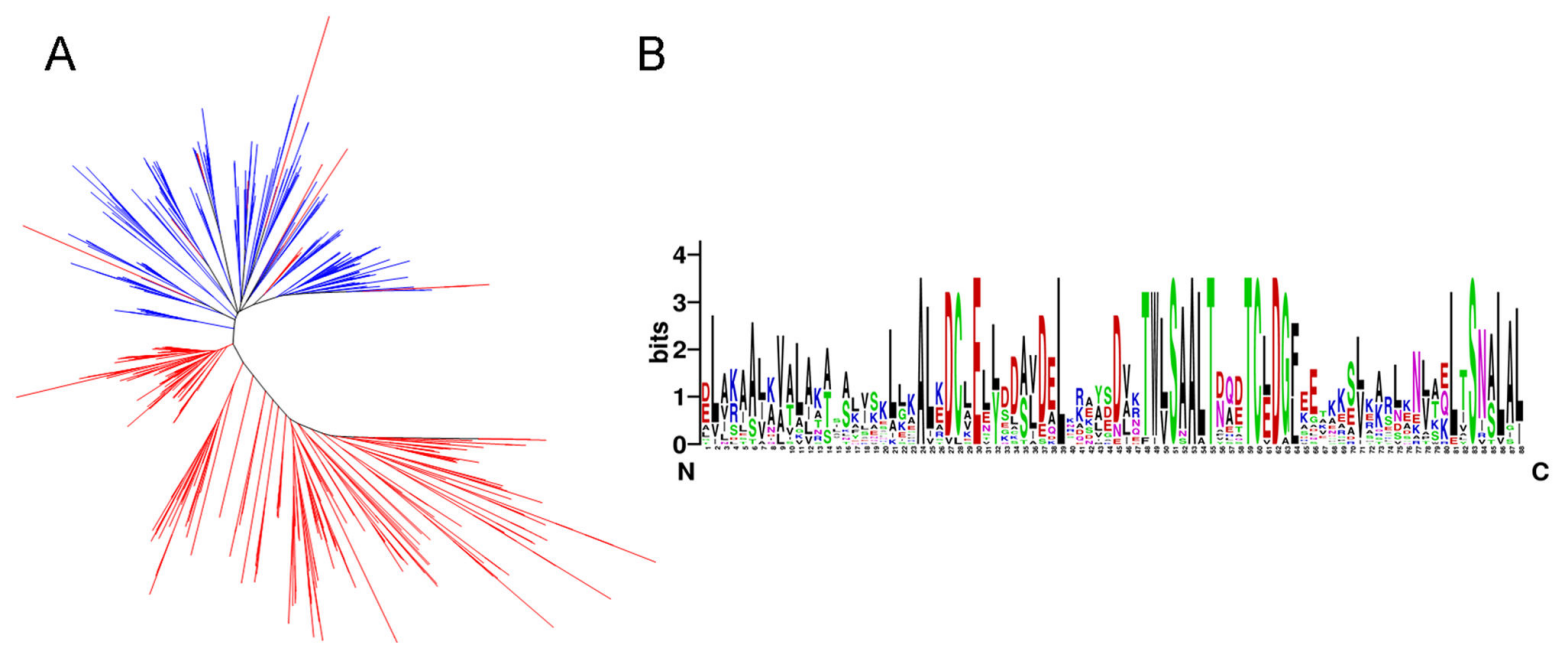
The molecular phylogenetic analysis of the orthologous PME genes, and pro and PMEI domains. (A) The phylogenetic analysis of the pro and PMEI domains in 10 species using the neighbor joining method. The blue lines represent the pro domain, and the red lines represent the PMEI domain. (B) The consensus sequence alignment shows the conserved motifs of the pro and PMEI domains in the representative species. The MEME program was then used to verify the conserved motifs.

The phylogenetic analysis was conducted to study the evolutionary relationship of the pro and PMEI domains. Firstly, the neighbor-joining tree of the pro and PMEI domains of all the representative species shows that they are obviously clustered into two clades, except the 16 PMEI domains from 8 noncommelinid species are classified into the pro domain clade (Figure 4A). To verify this result, the HMMER package was then used to construct consensus sequences of the pro and PMEI domains in every species by selecting the maximum probability residue at each match state. The phylogenetic analysis reveals that the consensus domain tree is not completely in accord with the species tree (Figure S4). The main differences include the species divergence between *P* *. patens* and *S* *. moellendorffii* in the pro domain clade and the divergence between *A* *. trichopoda* and *V. vinifera* in the PMEI domain clade. Although the two domains are attributed to each clade, further sequence alignment shows that they shared three conserved motifs, AL[KE] DCLEL[LY] [DS]D[AS] [VL] DELK, TW[LV] SAALT[DN] [QA] [DE] TC[LE] DG[FL] and LTSN[AS] LAL (Figure 4B), which indicates that pro and PMEI domains may have similar evolutionary origin.

### Strong Selection of the pro and PMEI Domains

**Figure 5:**
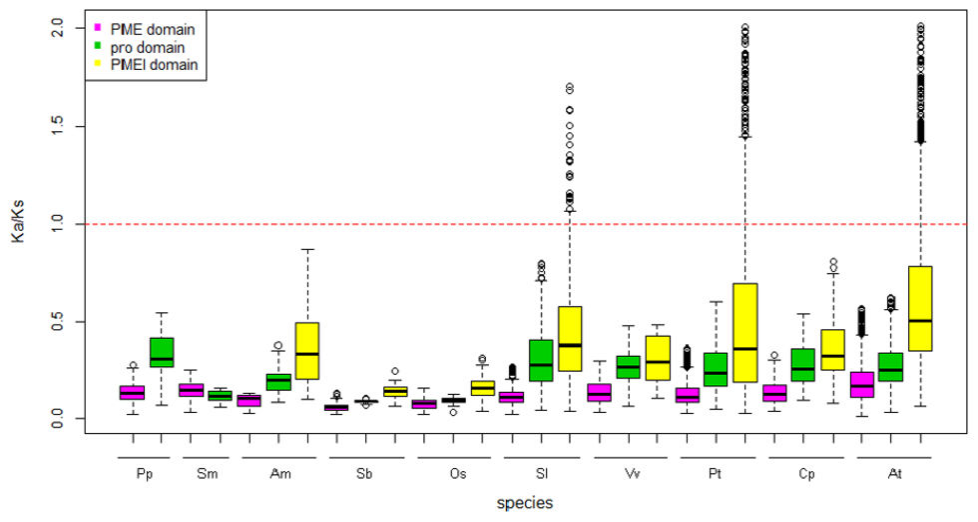
The Ka/Ks value distributions of the PME, pro and PMEI domains in 10 species. The red broken line indicates that genes are under positive selection (more than one) or negative selection (less than one). The short species names are *P* *. patens* (Pp), *S* *. moellendorffii* (Sm), *A* *. trichopoda* (Am), *S.* bicolor (Sb), *O. sativa* (Os), *S* *. lycopersicum* (Sl), *V. vinifera* (Vv), *P* *. trichocarpa* (Pt), *C. papaya* (Cp) and *A. thaliana* (At). The Ka and Ks values were computed using the PAML program.

To assess the potential selective forces of the evolving domains, the PAML software was used to calculate the Ka and Ks values of each pair in the three domains. The result of Ka/Ks analysis reveals that the PME and pro domains experienced purifying selection, but the pro domain showed stronger selection than the original PME domain (Figure 5). However, a large portion of the PMEI domains in the eudicot species (tomato, *Arabidopsis* and poplar) experienced positive selection. We observed that most offspring of PMEI genes from the recent α and β WGD events experienced positive selection in *Arabidopsis* , implying that they were quickly expanding. In the monocot rice and sorghum, all three domains experienced purifying selection, thus indicating a relatively different evolutionary pattern between eudicots and monocots.

### Comparative Expression Profile Analysis between *Arabidopsis* and Rice

Through expression data mining of the PME and PMEI families in the public databases, we presented the expression profiles of 63 tissue samples of *Arabidopsis* and 27 tissue samples of rice. Based on hierarchical clustering, the expression patterns in *Arabidopsis* can be clustered into six groups. The genes in group A are mainly expressed in leaves, shoots and seeds, the genes in group B are mainly expressed in roots and seeds, the genes in group C are mainly expressed in roots, the majority of genes in group D and E are expressed in vegetable tissues, and the genes in group F are specifically expressed in flower-related organs [51] (Figure S5a). In addition, the results show that all six groups consist of the PME, proPME and PMEI genes, thus indicating that although there are different expression patterns in different tissues, the proportional distribution of the genes in the two families shows no obvious bias in any group. By contrast, the expression profile in rice shows that most genes of the three clusters are not expressed specifically in tissues (Figure S5b), which may reflect the differences of pectin composition in primary cell walls between the eudicots and monocots.

### A Network Analysis of the *Arabidopsis* PME and PMEI Families

**Figure 6:**
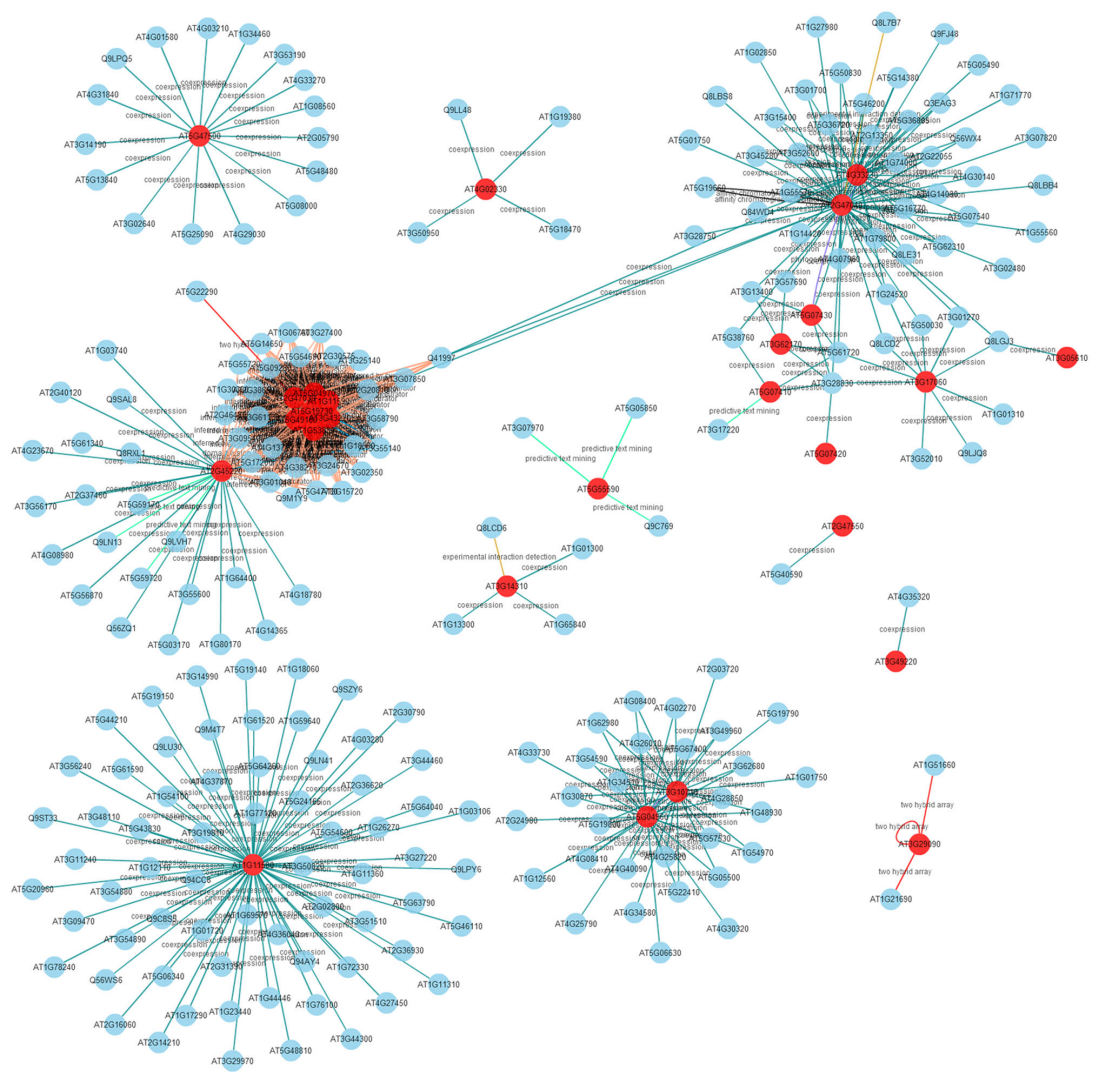
The *Arabidopsis* PME gene network. Twenty-six PME genes in *Arabidopsis* were mapped to the ANAP database. This network involves 257 unique genes exhibiting 514 interactions. The red nodes represent the PME genes.

Because the genes involved in a biological pathway typically express cooperatively [52], the PME and PMEI gene networks were constructed to further analyze the pectin metabolic system based on the pipeline of ANAP. Eventually, 26 of 67 PME genes in the ANAP database involves of 257 unique genes exhibiting 514 interactions, including co-expression and protein–protein interactions (Figure 6, Table S3). A further functional enrichment analysis reveals that these genes are mainly involved in the molecular function of transferase activity, ion binding, and hydrolase activity (Figure S6). In detail, among the 514 interactions, ADF11 and expansion A7 and A18 are directly related to cell wall formation [53,54], which obviously correspond to the function of the PME gene of pectin metabolism. Fourteen galacturonosyltransferases involved in pectin biosynthesis, and 16 pectin lyase-like genes involved in the degradation of demethyl-esterified pectin, are also detected in this network, thus indicating that complex pectin synthesis-degradation system related genes may act together temporally. Plant pathogen resistance genes are vital to plant development, and the network analysis also reveals that PME genes may function together with pathogen resistant proteins through the detection of 4 CAP (Cysteine-rich secretory proteins, Antigen 5, and Pathogenesis-related 1 protein) genes, consistent with the expression and network analysis of the pectin lyase-like gene family in *Arabidopsis* [55]. However, only 10 of 71 PMEI genes are present in the database, which consist of 34 interactions of 33 unique genes (Figure S7).

## Discussion

This study focused on the computational identification, gene duplication, evolution and functional analysis of the PME and PMEI families in fully or partially sequenced plant and algal genomes. We have found several important features of the relationship between the pectin demethyl-esterification process and the cell wall evolution.

### The Origin of the PME and PMEI Gene Families

Genome-wide gene identification has shown that PME genes can be identified after the appearance of pectins in cell walls (Figure 1). To improve the resolution of the evolutionary detection of the PME domain origin, we attempted to identify PME and PMEI gene families in chlorophyta (*C. reinhardtii* and *V. carteri*) [56,57], rhodophyta ( *C* *. merolae* ) [58], and diatoms ( *P* *. tricornutum* ) [59], but no such genes could be found. Although former research has identified PME genes in bacteria, the evolutionary relationship between land plants and bacteria remains unclear [60]. Therefore, we consider that plant PME genes may have originated from charophytes. Notably, this origin coordinates with the appearance of pectin in cell walls.

The identification of 15 PME genes in *C* *. orbicula* indicates that they appeared first evolutionarily in comparison with proPME and PMEI genes. The proPME appeared after the divergence of the bryophytes (Figure 1). These results suggest that PME genes occurred at an early stage of eukaryotes’ evolutionary history, and the domain fusion event between PME and pro domains possibly occurred after the divergence of charophytes. Compared with the PME and proPME genes, the PMEI family appeared later, most likely because of the gene neofunctionalization after the ζ and ε WGD events. In fact, the gene family synteny analysis shows that WGD and tandem duplication contributed to the expansion of the proPME and PMEI families in angiosperm species. Because both of PME and proPME genes possess the function of demethyl-esterification, further study should be carried to clarify the relationship between gene family expansion and the neofunctionalization of proPME genes. Our results indicate that there are two turning points of the pectin methyl-esterification process: the initial point was the divergence of moss from charophytes represented by the appearance of proPME, and the second point was the appearance of the PMEI family in land plants, which contributed to the complexity of the methyl-esterification process.

### A Putative Mechanism of PME, pro, and PMEI Domain Origin

Considering the sequence similarities between pro and PMEI domains, there must be some unclarifying evolutionary clues between them. Determining how the new pro domain emerged will be crucial to illustrate the evolutionary history of the PME and PMEI families.

Through the gene structure analysis, we observe that a large number of introns are lost after the domain fusion event by comparing the PME domain of proPME with the old PME genes (Figure S1). However, the origin of PME domain remains unknown. More comparative genomic studies should be performed with the genome sequencing of some important micro-algae. Notably, we find that the pro domains of two proPME genes in *Arabidopsis* (AT1G23200 and AT4G15980) show good sequence similarities with the introns of two original type II PME genes (AT1G44980 and AT3G27980). This observation may provide basic clues for an evolutionary interpretation of the pro domain origin (data not shown). The phylogenetic analysis and sequence alignment indicate that the PMEI domain may have originated from the pro domain, based on the sequence similarities, domain structures, and domain lengths and guided by genomic recombination or transposon-based recombination (Figure 4) [61,62]. The selection pressure analysis of the pro and PMEI domains indicates that they experienced strong positive selection, namely, they have rapidly evolved, particularly in tomato, poplar and *Arabidopsis* , in which lineage-specific whole genome duplication events have largely contributed to the expansion of the proPME and PMEI families (Figure 5).

Our model raised the possibility of domain origin through computer data mining, and additional research should be presented to further understand the evolution of the three domains. Meanwhile, an in-depth domain functional study can also clarify the evolutionary relationship of the pro and PMEI domains.

### Pectin Metabolism and Cell Wall Evolution

The origin and early evolution of the land plants provide good opportunities for research on plant cell wall evolution, although there are no very good structural models suggested currently [63]. New biophysical and visualization methods are necessary to understand the wall organization of components in a single cell [2,64]. A component analysis has summarized the basic metabolite appearances using uni- and multicellular algae, mosses, basal angiosperms, eudicots and monocots. Cellulose and cellulose syntheses genes originated at the divergence of rhodophyta from glaucophyta [65]. Homogalacturonan (HG), a polymer of pectin, is widely present in the cell walls of uni- and multicellular species, which is frequently the case in embryophytes and land plants. Rhamnogalacturonan II (RGII), also a polymer, was initially observed in embryophytes and the content generally increased during the evolution of vascular plants, which is a trend that may satisfy the formation of lignified secondary walls but not in the monocot species. PME and PMEI genes spatially regulate the methyl-esterification of pectin polymers. In fact, pectin polysaccharides are complex in the cell wall and involved in large families of related synthesis and degradation genes. In addition to the PME and PMEI families, the *Arabidopsis* genome also encodes approximately 65 glycosyltransferases [6], 67 methyltransferases, and 67 pectin lyases [57]. The complex pectin metabolism may be related to functional diversity. Research has shown that demethyl-esterification of pectins in cell walls is related to organ initiation in *Arabidopsis* [66]. In this study, the functional network also shows that PME genes may be involved in plant–pathogen interactions and affect plant resistance to diseases (Figure 6). This result may be related to the methyl-esterification of pectins during plant–pathogen interactions [67]. Although the exact pathway and interaction network mediated by these genes remains unclear, we can speculate on and expect these putative mechanisms for further functional study.

Overall, these results generate a new insight into the genic evidence of pectin divergence and how these genes evolved in algae and land plants from the perspective of bioinformatics. Meanwhile, expression profiling and functional network analysis in model species may help us better understand the possible molecular mechanisms of the primary cell wall biosynthesis.

## Supporting Information

Figure S1 **The exon-intron structural analysis of the proPME and PMEI family in *Arabidopsis* .**
The legend shows that the blue boxes are the UTR regions, the green boxes are exons, the black lines are introns, the red boxes are the PME, pro and PMEI domains, and the numbers at the exon-intron joints are the intron phases. (A) The exon-intron structural analysis showed the PME domain of the proPME. (B) The exon-intron structural analysis shows the pro domain of the proPME. (C) The exon-intron structural analysis shows the PMEI domain of the PMEI in *Arabidopsis* .
(TIF)

Figure S2 **The consensus sequence alignment showed the conserved motifs of the PME domain in representative species.**
HMMER package was used to trim the consensus sequence of the PME domain in the eleven species, and the NOGAPS sequence alignment was retained. MEME program was used to validate the conserved motifs.
(TIF)

Figure S3 **The molecular phylogenetic analysis of the orthologous PME genes from eleven species.**
The ortholog gene cluster was initially identified from the output of Orthomcl software and verified by a subsequent single cluster phylogeny analysis. The molecular clock test was performed by comparing the ML value for the given topology with and without the molecular clock constraints under the WAG model. The null hypothesis of an equal evolutionary rate throughout the tree was rejected at a 5% significance level. The evolutionary analysis was conducted in MEGA5.
(TIF)

Figure S4 **The molecular phylogenetic analysis of the pro and PMEI domains using the maximum likelihood method.**
The evolutionary history was inferred by using the Maximum Likelihood method based on the WAG model with 500 replications for the bootstrapping test. Each branch represents the consensus domain sequences trimmed using HMMER.
(TIF)

Figure S5 **Expression profiling of the PME, proPME and PMEI families in *Arabidopsis* and rice.**
The uppercase-lowercase ‘At’ and ‘Os’ are the PME genes, the lowercase-lowercase ‘at’ and ‘os’ are the proPME genes, and the uppercase-uppercase ‘AT’ and ‘OS’ are the PMEI genes. (a) The co-expression profile of *Arabidopsis* PME and PMEI families in 63 tissue samples. These genes were divided into 6 groups using the complete linkage clustering method. (b) Co-expression profiling of the rice PME and PMEI families in 27 tissue samples. The M indicates rice variety Minghui 36, and Z is variety Zhenshan 97.
(TIF)

Figure S6 **Functional enrichment of the genes involved in the *Arabidopsis* PME gene network.**
The GO enrichments were performed with Blast2GO software, and the molecular function category of level three was analyzed.
(TIF)

Figure S7 **The *Arabidopsis* PMEI gene network.**
This network involves 34 unique genes exhibiting 33 interactions. The red nodes represent the PMEI genes.
(TIF)

Table S1 **The genome-wide identification of the PME, proPME and PMEI genes.**
This table shows genes from 11 species, *C* *. orbicular* (Co), *P* *. patens* (Pp), *S* *. moellendorffii* (Sm), *A* *. trichopoda* (Am), *V. vinifera* (Vv), *C. papaya* (Cp), *P* *. trichocarpa* (Pt), *A. thaliana* (At), *S.* lycopersicum (Sl), *O. sativa* (Os) and *S. bicolor* (Sb).
(XLSX)

Table S2 **The identified orthologous groups of the PME family genes in 11 representative species.**
The orthologous genes were defined as genes in a cluster from at least three species. This analysis was conducted using Orthomcl software.
(XLSX)

Table S3 **The annotation summary of the genes involved in the PME network.**
The annotation information was downloaded from TAIR (The *Arabidopsis* Information Resource, http://www.arabidopsis.org/).
(XLSX)
