# Supplementary figures and images for: A Comparative Genome Analysis of PME and PMEI Families Reveals the Evolution of Pectin Metabolism in Plant Cell Walls
Source: PLoS One. 2013 Aug 12;8(8):e72082. doi: 10.1371/journal.pone.0072082 (PMC3741192; doi:10.1371/journal.pone.0072082)

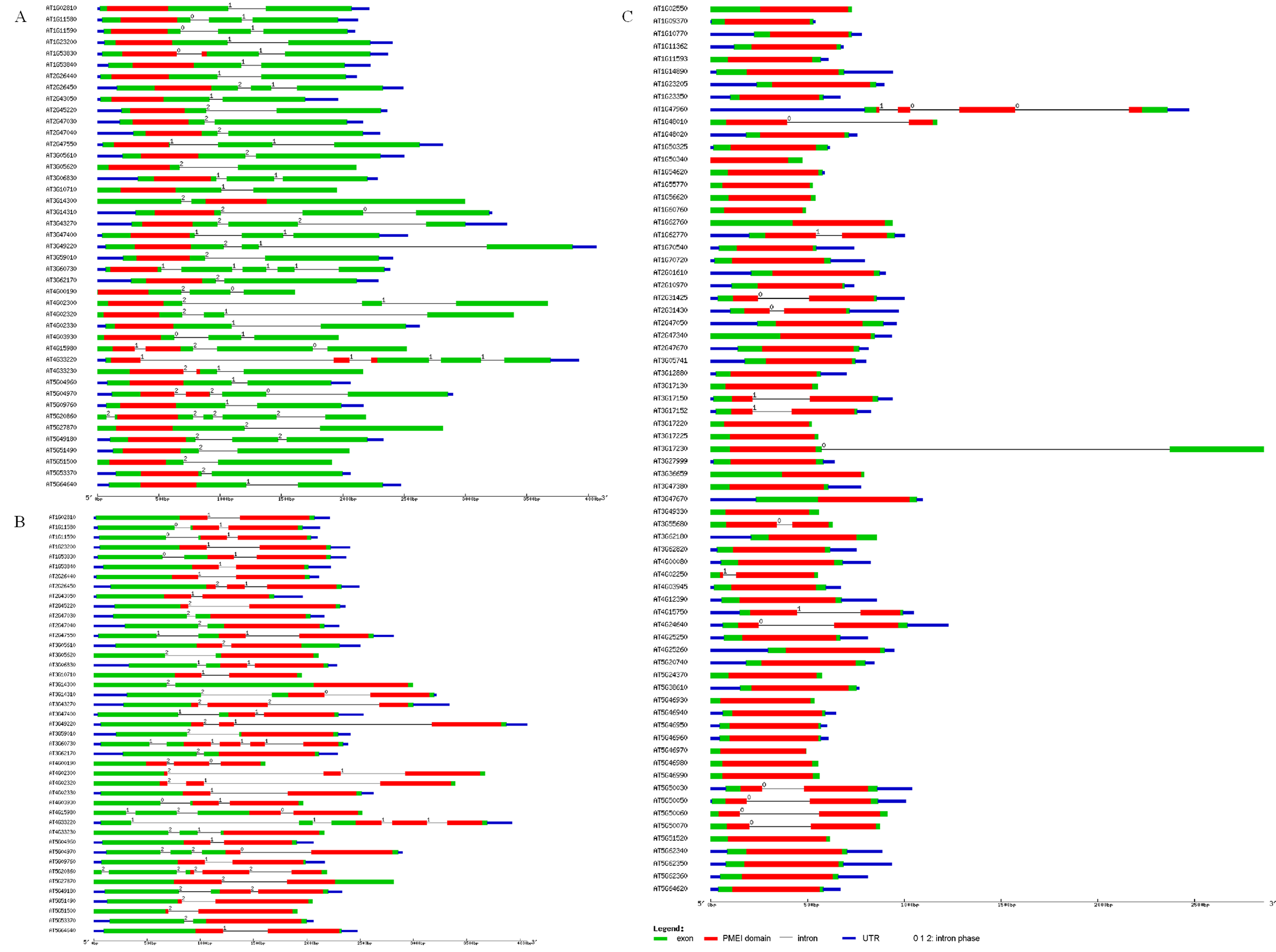

Supplement: Figure S1 — The exon-intron structural analysis of the proPME and PMEI family in Arabidopsis . The legend shows that the blue boxes are the UTR regions, the green boxes are exons, the black lines are introns, the red boxes are the PME, pro and PMEI domains, and the numbers at the exon-intron joints are the intron phases. (A) The exon-intron structural analysis showed the PME domain of the proPME. (B) The exon-intron structural analysis shows the pro domain of the proPME. (C) The exon-intron structural analysis shows the PMEI domain of the PMEI in Arabidopsis . (TIF) [file pone.0072082.s001.tif]

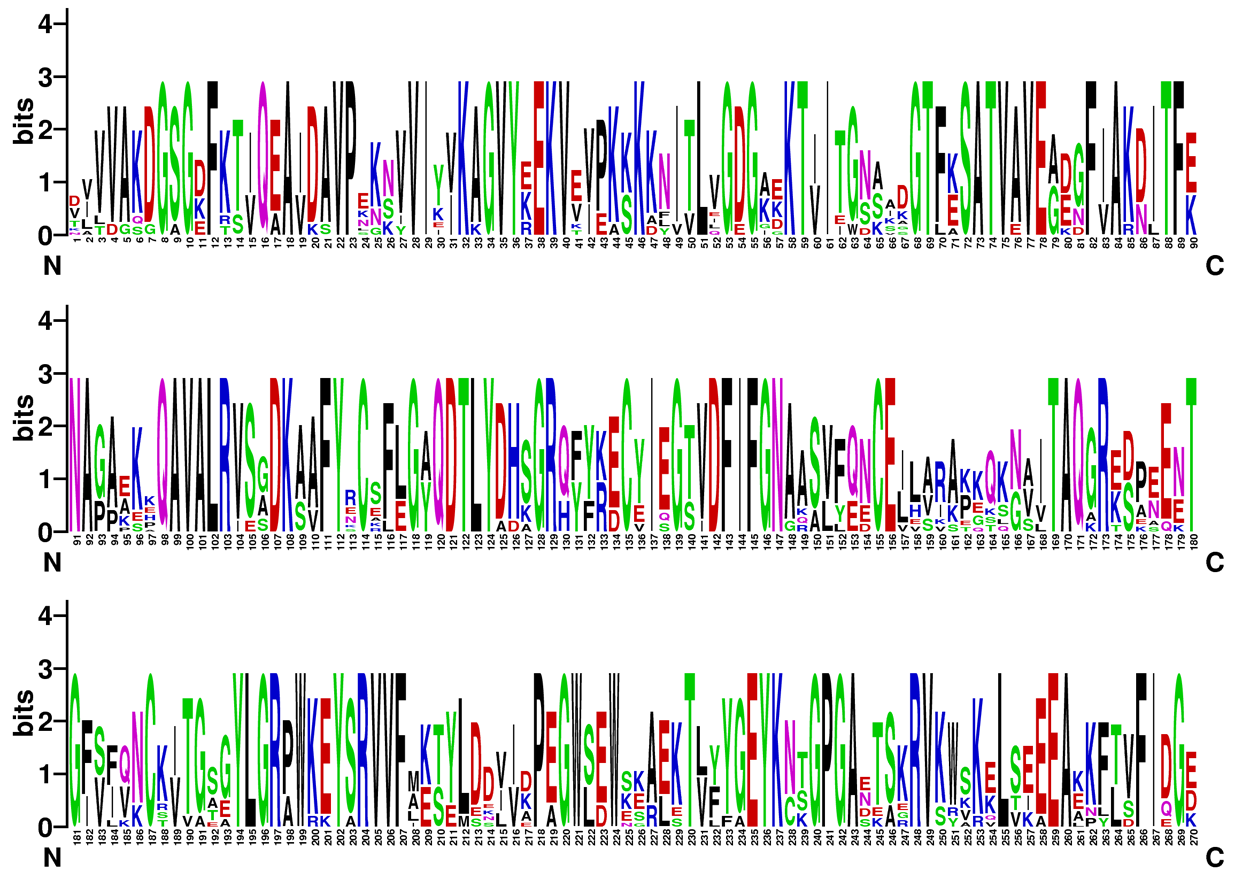

Supplement: Figure S2 — The consensus sequence alignment showed the conserved motifs of the PME domain in representative species. HMMER package was used to trim the consensus sequence of the PME domain in the eleven species, and the NOGAPS sequence alignment was retained. MEME program was used to validate the conserved motifs. (TIF) [file pone.0072082.s002.tif]

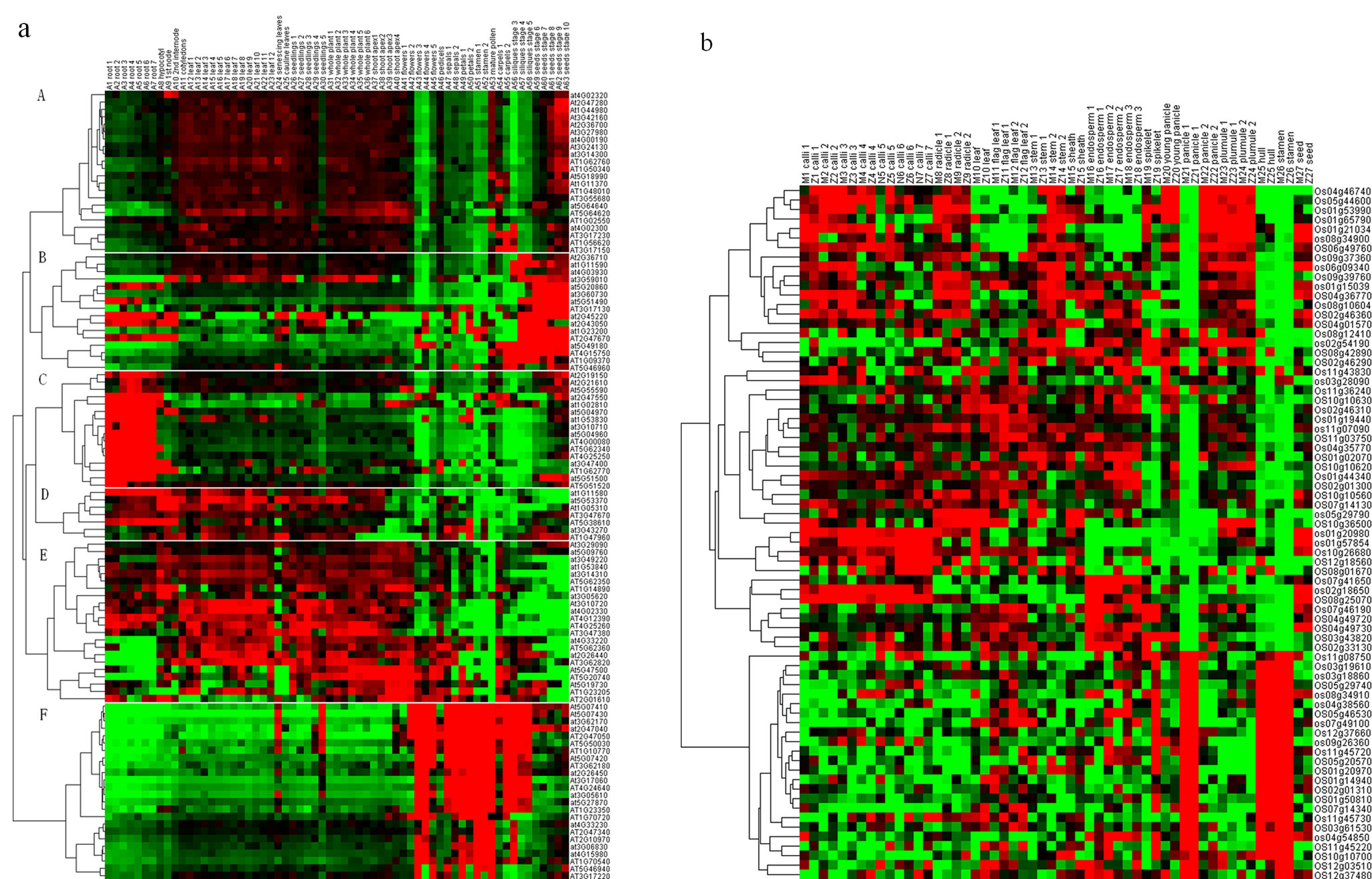

Supplement: Figure S5 — Expression profiling of the PME, proPME and PMEI families in Arabidopsis and rice. The uppercase-lowercase ‘At’ and ‘Os’ are the PME genes, the lowercase-lowercase ‘at’ and ‘os’ are the proPME genes, and the uppercase-uppercase ‘AT’ and ‘OS’ are the PMEI genes. (a) The co-expression profile of Arabidopsis PME and PMEI families in 63 tissue samples. These genes were divided into 6 groups using the complete linkage clustering method. (b) Co-expression profiling of the rice PME and PMEI families in 27 tissue samples. The M indicates rice variety Minghui 36, and Z is variety Zhenshan 97. (TIF) [file pone.0072082.s005.tif]

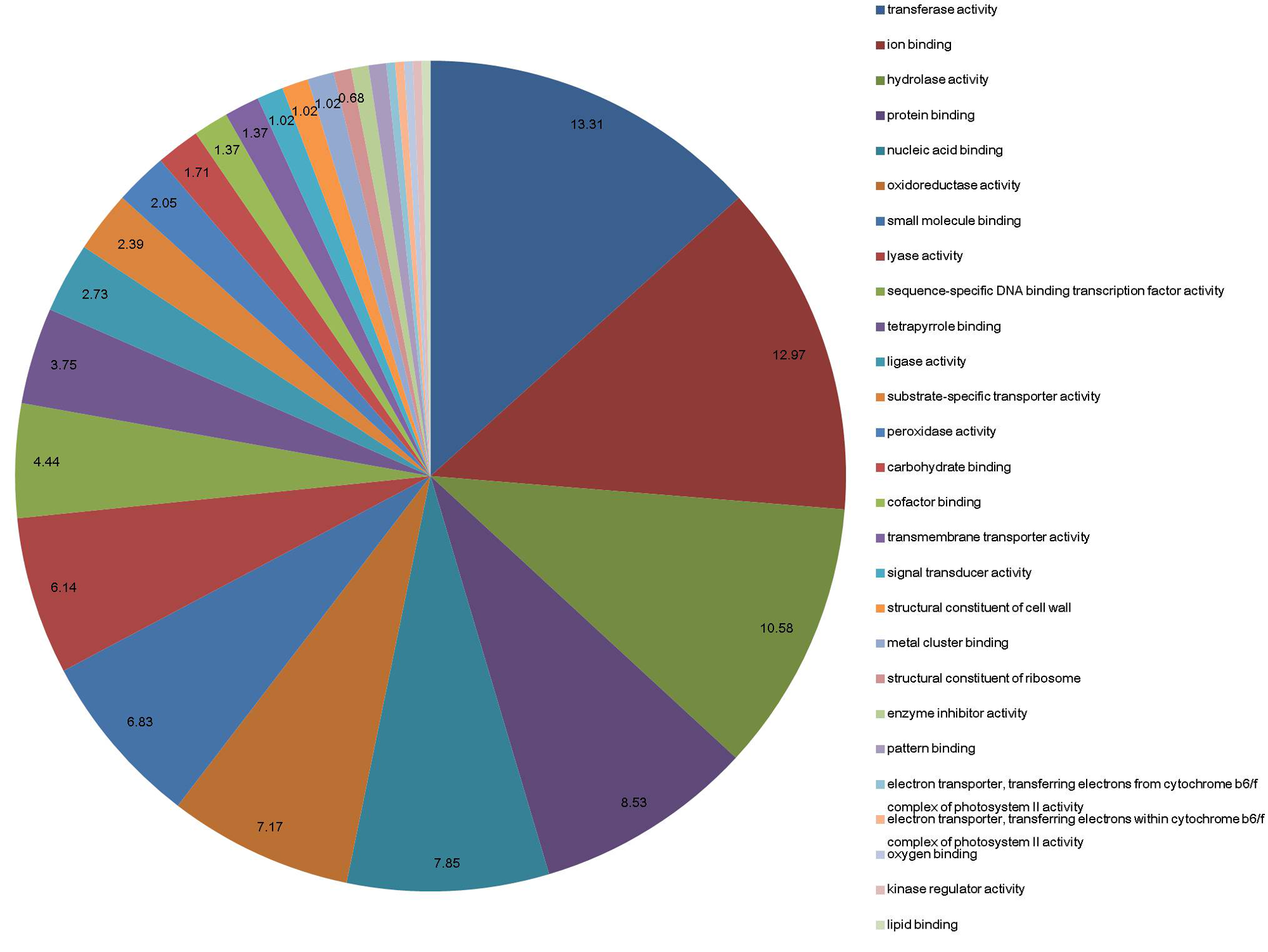

Supplement: Figure S6 — Functional enrichment of the genes involved in the Arabidopsis PME gene network. The GO enrichments were performed with Blast2GO software, and the molecular function category of level three was analyzed. (TIF) [file pone.0072082.s006.tif]

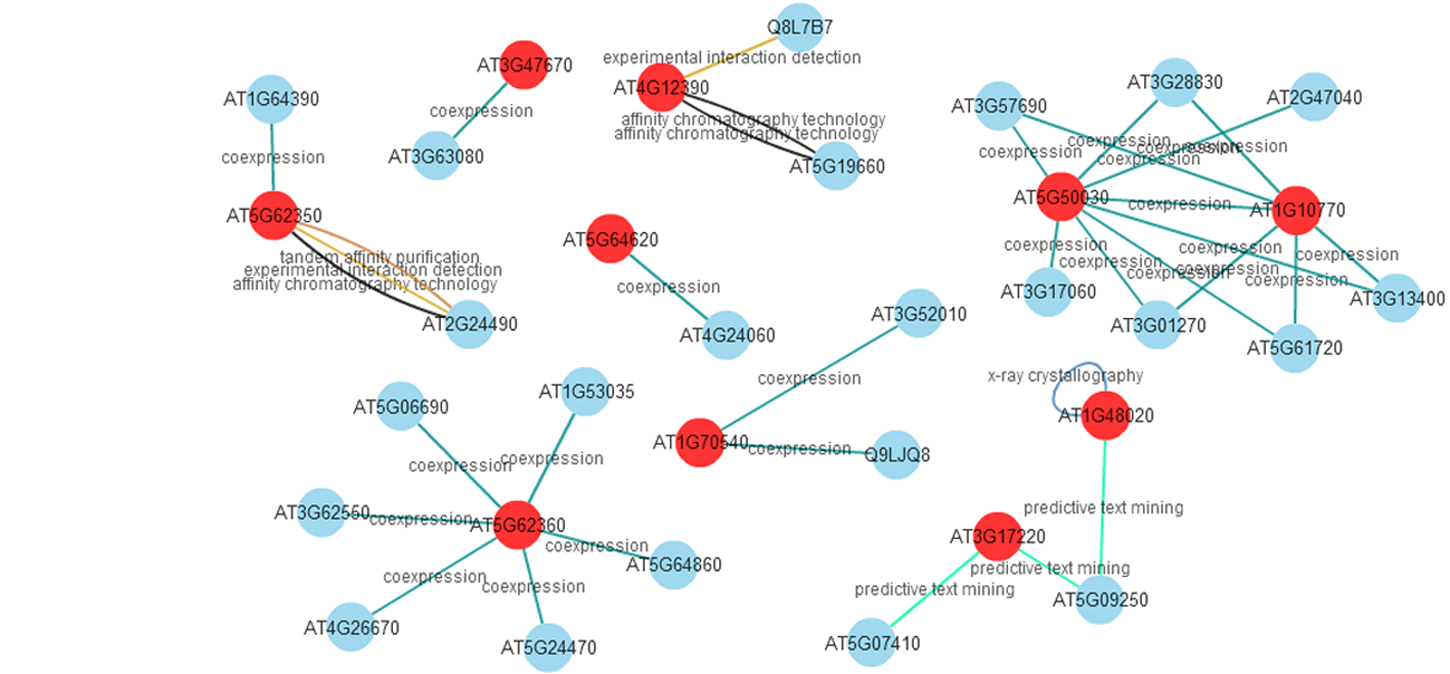

Supplement: Figure S7 — The Arabidopsis PMEI gene network. This network involves 34 unique genes exhibiting 33 interactions. The red nodes represent the PMEI genes. (TIF) [file pone.0072082.s007.tif]
